# An Estimate of Global Anthrax Prevalence in Livestock: A Meta-analysis

**DOI:** 10.14202/vetworld.2021.1263-1271

**Published:** 2021-05-22

**Authors:** Bylaiah Sushma, Seema Shedole, Kuralayanapalya Puttahonnappa Suresh, Gowda Leena, Sharanagouda S. Patil, Gowda Srikantha

**Affiliations:** 1 Department of Computer Science and Engineering, M S Ramaiah Institute of Technology, Matthikere, Bengaluru, Karnataka, India; 2 Spatial Epidemiology Laboratory, Indian Council of Agricultural Research (ICAR) National Institute of Veterinary Epidemiology and Disease Informatics (NIVEDI), Yelahanka, Bengaluru, Karnataka, India; 3 Department of Veterinary Public Health and Epidemiology, Veterinary College, Hebbal, Bengaluru, Karnataka, India; 4 Virology Laboratory, Indian Council of Agricultural Research (ICAR) – National Institute of Veterinary Epidemiology and Disease Informatics (NIVEDI), Yelahanka, Bengaluru, Karnataka, India

**Keywords:** anthrax, livestock, meta-analysis, prevalence, subgroup analysis

## Abstract

**Background and Aim:**

Anthrax, caused by the soil-borne spore-forming bacteria called *Bacillus anthracis*, is a zoonotic disease that persists worldwide in livestock and wildlife and infects humans. It is a great hazard to livestock; henceforth, evaluating the global concerns about the disease occurrence in livestock is essential. This study was conducted to estimate the global prevalence of anthrax and predict high-risk regions, which could be an input to veterinarians to take necessary steps to control and avoid the disease.

**Materials and Methods:**

A literature review was performed using online databases, namely, PubMed, Google Scholar, Scopus, Biomed Central, and Science Direct, to extract relevant publications worldwide between 1992 and 2020.</AQ9> Initially, 174 articles were selected, and after scrutinizing, 24 articles reporting the prevalence of anthrax were found to be adequate for the final meta-analysis. The statistical study was accompanied by employing fixed effects and random effects models using R.

**Results:**

The pooled prevalence of anthrax globally was 28% (95% confidence interval, 26-30%) from 2452 samples through the fixed effects model. Continent-wise subgroup analysis through the random effects model revealed that the pooled prevalence of anthrax was highest in Africa (29%) and least in North America (21%).

**Conclusion:**

In these publications, anthrax causes economic loss to farmers and, thus, to the world. Hence, controlling anthrax infections in high-risk regions are essential by implementing appropriate control measures to decrease the effect of the disease, thereby reducing economic loss.

## Introduction

Anthrax is an ancient and severe disease that causes the loss of livestock. Besides, human beings commonly get infected through contact with infected animals and their products or through working exposure in clinics and agricultural fields. Anthrax is a bacterial infection caused by *Bacillus anthracis*, an aerobic, Gram-positive spore-forming bacterium and occurs primarily as a cutaneous, pulmonary, or gastrointestinal infection, depending on the route of entry of *B. anthracis* spores [1]. *B. anthracis* mostly affects grazing animals, namely, cattle, sheep, and goats that can be infested by consuming spores existing in contaminated soils. Gnawing flies might be associated with disease transmission in certain areas [2]**.** The spore form of *B. anthracis* in the soil turns inactive and continues for a long time; such that, years may pass between outbreaks. Natural conditions contribute to its transmission, such as higher soil type, calcium levels, increased temperature and humidity, slightly alkaline pH, and higher levels of decaying organic matter, along with the organism’s ability to live in a harsh environment for extended survival of the spore in the soil. Moreover, the spore is resistant to sunlight, heat, drying, and many disinfectants [3]. Initially, researchers have suggested that these factors influence vegetative anthrax bacilli. Nonetheless, a study has demonstrated that the vegetative cells of *B. anthracis* have certain supplemental and physiological necessities and are improbable to survive outside the host. A survey of the properties of *B. anthracis* spores and different *Bacillus* species has recommended that the particular soil factors connected to pandemic regions reflect significant ecological conditions that guide *B. anthracis* spores in causing ailments. Particularly, significant levels of calcium in the soil may assist with keeping up spore essentialness for delayed periods, along with the opportunity of spores experiencing and contaminating another host [1]. When the bacterium invades living organisms, it germinates and starts replicating in the freshly infected animal. Henceforth, control actions are vital to the survival of the livestock populace. The top and globally accepted cost-effective control measures of infection in livestock are annual vaccination and active surveillance program to recognize outbreaks early in the epidemic progression. Besides, the proper disposal of carcasses is crucial for the prolonged endurance of the agent in the soil. The corpses of infected animals should be predisposed without opening to avoid sporulation and further contamination of the soil [3].

Anthrax is global in its geographical distribution and is endemic to many parts of South Europe, Asia, Africa, North and South America, and Australia [1]. This study analyzes the worldwide prevalence of anthrax using a meta-analysis. Meta-analysis is a broadly extending field of research and a formal, quantitative, epidemiological examination used to deliberately survey explorative publications to determine decisions about a huge volume of information. Meta-analyses that are well-guided may act as an important tool for increasing animal productivity and prosperity. Arranging disclosures from various examinations are needed to ensure that meta-analytic exploration is appealing. A huge measure of publication discoveries has been made in animal prosperity and production, making meta-analytic studies more promising [4]. The meta-analysis steps include defining the domain of research and hypothesis; defining consideration/avoidance rules; searching for historical data; choosing the finalized publications; extricating information on factors of interest, coding systems, figuring impact sizes, and understandings; choosing expected moderators; analyzing their relationships; and writing report and critical assessment results [5]. Subgroup analysis includes parting all participant information into subgroups to examine the relationships between them. Subgroup analysis might be accomplished for subsets of publications, for example, different geological areas. Subgroup analysis could be conducted as a method for exploring diverse outcomes.

This study was conducted to estimate the global prevalence of anthrax and predict high-risk regions, which could be an input to veterinarians to take necessary steps to control and avoid the disease.

## Materials and Methods

### Ethical approval

Ethical approval was not necessary as we have not collected any animal samples for the study.

### Literature search

A methodical investigation was performed on the literature about the worldwide prevalence of anthrax in livestock. The information was pooled from the following databases: PubMed, Google Scholars, Science Direct, Biomed Central, and Scopus. Studies reporting the prevalence of anthrax were systematically reviewed and included for meta-analysis. More than a thousand articles were looked at, assessed, and chosen, and the outcomes were exposed to meta-analysis to decide the prevalence rate of anthrax concerning different periods and different continents. The literature search from various publications was embraced for the period from 1992 to 2020. Given a huge quantity of literature, we assembled and consolidated the attributes of the publications, such as author, publication year, continent, number of samples tested, number of positive samples, and animals (i.e. species, such as cattle, sheep, goats, and buffalo). The recovery language was restricted to English. Original articles, peer-reviewed articles, and references cited from the retrieved articles were examined again to follow the previous year’s publications. The preferred reporting items for systematic review and meta-analysis protocol (http://www.prisma-statement.org) were followed in conducting the study.

### Study selection and data extraction

The publications were confined to studies on the prevalence of anthrax in cows, sheep, goats, and buffalo species worldwide. The gathered articles were completely inspected for replications and were eliminated. The additional standards to consider for meta-analysis are the number of animals tested, number of animals infected, time of study, and publications that have used the standardized confirmatory test, for example, blood smear examination, staining techniques, sero-analysis by enzyme-linked immunosorbent assay (ELISA), and nucleic acid-based methods by polymerase chain reactions (PCRs). The publications referring to outbreak assessments, survey articles, case reports, and clinical primers were dismissed from the examination. Relevant studies were taken in light of the aforementioned standards, and the outcomes from the discrete examinations were pulled out freely to a predesigned data collection sheet. Information extricated from the chosen publications were as follows: The study year, sample size, number of animals positive for *B. anthracis*, method used for diagnosis, author’s name, study location, and publication year. The estimation of the prevalence of anthrax for individual studies was intended to choose the uppermost prevalence when various diagnostic methodologies were employed. The stages included for efficient data filtering were as follows: (i) The analysis method used was lucid with the goals of the meta-analysis, and its goals directed a few qualities to be estimated and stated as reports. (ii) Guaranteeing that a chosen study does not have an anomaly concerning quality and relations are essential.

### Meta-analysis

Meta-analysis for publications on prevalence was performed to generate a weighted average ratio of the prevalence of anthrax in numerous publications, allowing us to acquire a more precise measure of anthrax prevalence from several publications, consequently giving a superior heading toward imminent work [6]. Precise reviews were used to incorporate discoveries from accessible research publications at the highest quality level to estimate the viability of preventive and restorative mediations for the predetermined settings. The meta-analysis of anthrax prevalence in livestock was conducted using R (version 3.4.3; R Foundation for Statistical Computing, Vienna, Austria; https://www.R-project.org/). The packages of R used for meta-analysis were as follows: *metafor, meta*, and *qdap*. Forest plots were used to visually represent the meta-analysis. Forest plots show the impact gauge and confidence intervals (CIs) of each examination. Every study is depicted as a square at a point estimate of impact, and a line depicted horizontally broadening both sides of the square portrays a 95% CI. The square area corresponds to the weight assigned to that review in the meta-analysis. The impact of the model was picked relying on the level of heterogeneity (*I^2^*). The most extreme probability assessor was used to decide between-study variances *τ^2^*. Since generous heterogeneity was normal, fixed effects and random-effects models were used to reveal a pooled prevalence of anthrax.

### Publication bias

The chances of publication bias were measured using a funnel plot with the Y-axis representing the standard error of each study and the X-axis representing the Arcsine transformation of the proportion of each study. If the publication bias is nil, high-accuracy investigations lie along the line of normal, whereas low-accuracy investigations scatter equitably on both margins of the normal line, making a funnel-shaped distribution [7]. The dispersion of publications in the funnel formation directs to publication bias. Furthermore, funnel plot deflection was curbed using the rank correlation strategy, Egger’s test, and the linear regression test. The p-value of every null hypothesis test was either rejected or accepted. The Trim and Fill strategy was used to regulate the funnel plot deviation. To decide the level of discrepancy over multiple publications due to heterogeneousness as opposed to risk, the Cochran Q test (Chi-square test of heterogeneity) and heterogeneity statistic *I^2^* (Higgins *I^2^*) were determined. To measure the heterogeneity, *I^2^* estimations of 25%, 50%, and 75% were used to denote low, medium, and high heterogeneity, respectively [8]. The H value was computed to encapsulate the effect of heterogeneity. Subsequently, the H statistic, without maximum boundary, will permit fluctuations in heterogeneity with great validness when the quantities of publications are fewer.

### Subgroup analysis

Subgroup analysis is the procedure where all participant data incorporated in the meta-analysis are divided into subgroups, according to trial characteristics, namely, geographical location, or patient characteristics, namely, gender, and then a meta-analysis is performed on one or more of these subsets. Sources of heterogeneity can be found by conducting a subgroup analysis [9]. In this study, subgroup analysis was conducted to assess the heterogeneity among publications from different continents. The impact of the model was selected relying on the level of heterogeneity (*I^2^*). The most extreme probability assessor was used to decide between-study variances *τ^2^*. A random-effects model was used to show a pooled prevalence of anthrax from a global perspective.

### Statistical analysis

A two-stage methodology was adopted in the statistical aspects of a meta-analysis. The summary statistics from each analysis were determined in the first level. The summary statistics from each sample were combined in the second level to provide an aggregate result. We performed the first level with the help of Rayyan systematic review and Zotero software. Second level was conducted by employing fixed effects and random-effects meta-analysis. A fixed-effect meta-analysis assumes that all observed variance was due to chance, i.e., sampling error within the sample. The random-effects model, on the other hand, allows for differences between experiments. Since the random vs. fixed-effect model is generally the appropriate model, meta-analysis was conducted using fixed effect and random effects model and subgroup analysis was conducted by employing random effects model in the present study to estimate global anthrax prevalence.

## Results

### Details of prevalence publications

**Figure-1:**
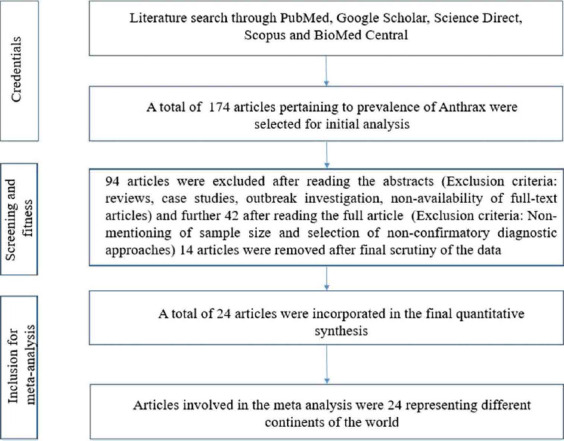
Schematic diagram showing the literature search with exclusion/inclusion procedure for meta-analysis.

The online database searches returned 2875 likely articles related to the keyword search. Review publications on the prevalence of anthrax in humans were omitted. After the initial scrutiny of the titles of eligible articles, those that have reported the prevalence of anthrax were selected, whereas those that were irrelevant were excluded from the study. Subsequently, 174 articles were retained after the initial assessment. Among them, after analyzing the abstracts, 94 were removed, and a further 42 articles were removed by subsequently analyzing the full article. Thirty-eight publications were selected, and after the final data scrutiny, 24 articles were included in the meta-analysis. The addition and omission benchmarks followed for the meta-analysis of the studies on the prevalence of anthrax are presented in Figure-1.

### Meta-analysis of anthrax prevalence in livestock

**Figure-2:**
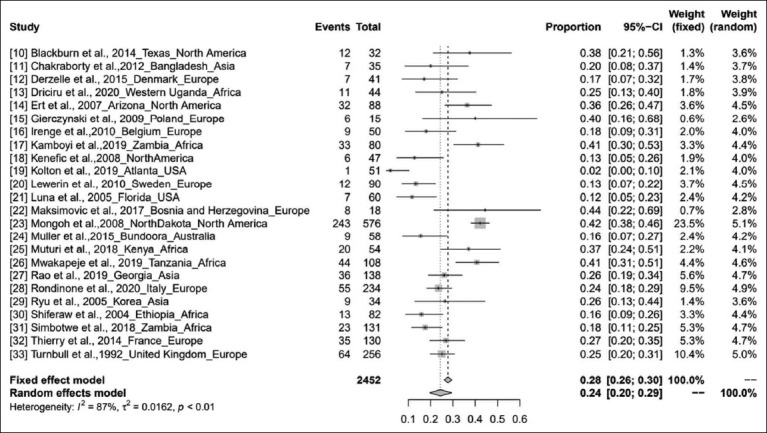
Forest plot showing the results of meta-analysis.

This study covered five continents: Africa, Asia, North America, Europe, and Australia. No suitable publications were available for South America. The number of publications included in the meta-analysis was 24, including 2452 samples, from 1992 to 2020. The meta-analysis designated that inconsistency was more among the publications (*τ^2^*=0.0162; heterogeneity *I^2^*=87%; heterogeneity variability *H^2^*=7.11; p<0.01). The pooled prevalence by the fixed effects models was 28% (95% CI, 26-30%). The pooled prevalence by the random-effects model was 24% (95% CI, 20-29%) (Figure-2) [10-33].

**Figure-3:**
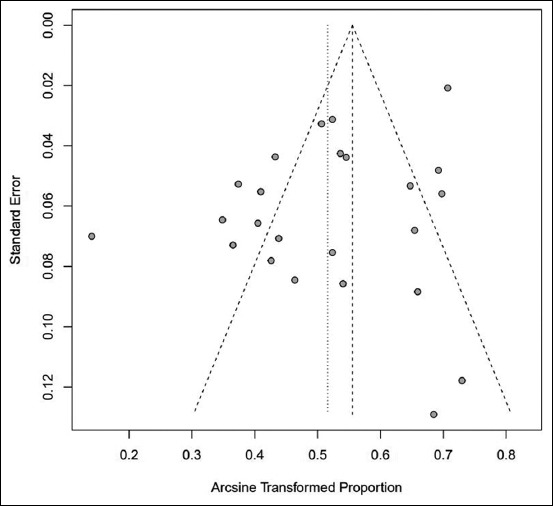
Funnel plot showing publication bias.

Asymmetry of the funnel plots was found using the Begg and Mazumdar rank correlation test, with the results showing that Kendall’s *tau* was 0.08 with a p=0.61, and the Eggers regression test using a fixed-effects meta-regression model, with the results showing that *z* was −5.28 with p<0.01. The results demonstrated generous deviation in the funnel plots (rejected the null hypothesis), hence uncovering the probable occurrence of publication bias (Figure-3). Inconsistency, heterogeneity, and publication bias were observed among the included publications due to which further subgroup analysis was conducted.

### Subgroup meta-analysis

**Figure-4:**
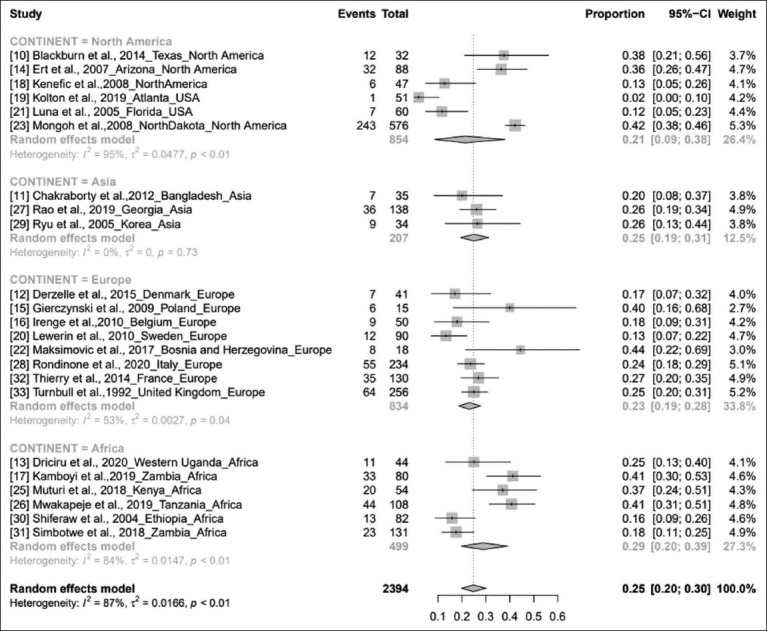
Forest plot showing anthrax prevalence in different continents.

**Figure-5:**
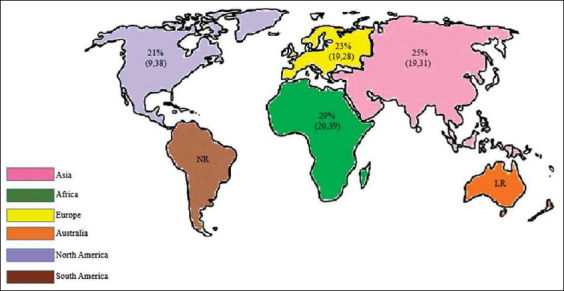
World map showing the continent-wise prevalence estimates of anthrax based on subgroup meta-analysis. NR: No reports, LR: Limited reports [Source; Raw map sourced from https://d-maps.com/index.php?lang=en}.

A continent wise study showed that the pooled prevalence of anthrax for the period from 1992 to 2020 in Africa was 29% (95% CI, 20-39%; *I*^2^=84%; *τ^2^*<0.0147; p<0.01), followed by Asia (25%; 95% CI, 19-31%; *I*^2^=0%; *τ^2^*=0; p=0.73), Europe (23%; 95% CI, 19-28%; *I*^2^=53%; *τ^2^*=0.0027; p=0.04), and North America (21%; 95% CI, 9-38%; *I*^2^=95%; *τ^2^* 0.0477; p<0.01). Nevertheless, the subgroup analysis showed an overall prevalence of 25% (95% CI, 20-30%; *I^2^*=87%; *τ^2^*= 0.0166; p<0.01), and the residual heterogeneity *I^2^* was 87% (p<0.01) by the random effects model (Figure-4) [10-33]. The world map of continent wise pooled prevalence of anthrax in livestock based on the results of the subgroup meta-analysis is shown in Figure-5.

## Discussion

Anthrax is a globally distributed disease and is endemic to many parts of Africa, Asia, South Europe, North and South America, and Australia, which decelerates the progress and sustainability of the livestock sector [34]. In general, anthrax outbreaks occur during the hot and humid transition period between the dry and wet seasons. During this period, soil temperatures are higher than usual in both day and night. The soil is also significantly disturbed during the cultivation season, which contributes to the multiplication of bacteria, increasing subsequent soil-borne infections of animals. Shiferaw *et al*. have considered Wabessa village from Dessie Zuria district in Ethiopia for the study [30]. The virulence of the *B. anthracis* strains is followed by two large plasmids, pXO1 and pXO2, and strains lacking either plasmid will become virulent or substantially weakened [35]. Disease spread in populations is a consequence of the interaction between host, pathogen, and environment, that is, the epidemiological chord. Yet, the effects of each chord component may vary dramatically in various settings [36]. Worldwide, domestic cattle are the most commonly reported livestock with anthrax [10]. The anthrax immunochromatographic test is considered the best diagnostic test for samples taken from animals suspected to have anthrax within 48 h of death, and this study showed a prevalence of 24% (95% CI, 19-30%) [11]. Standard single-nucleotide polymorphism (SNP) composing and entire genome sequencing were used to examine the subatomic variety of *B. anthracis* strains secluded from cows [12]. The Active Anthrax Detect Rapid Test parallel stream insusceptible test is a state-of-care test that was under investigational use for recognizing *B. anthracis* [19]. The earliest possible awareness of an anthrax attack could reduce illness and death. A progressing challenge to the observation approach is that no exact clinical calculation exists to openly recognize whether a bacterium is separated from blood culture or culture infection [37]. Although robust techniques to test the positivity of *B. anthracis* are lacking, certain studies have shown different methodologies for disease detection.

An ongoing PCR test was produced for the quick identification of *B. anthracis* [16,20,38]. Field arranged blood smears were verified by microscopy using four recoloring strategies, including PCR trailed by Bayesian latent class investigation [39]. Samples were tested using a 31-marker multilocus variable-number tandem repeat analysis (MLVA) to identify different genotypes. Different genotypes separated from the same animal could result either from freshly rising mutations during the incubation period of the disease or through mixed infections. The latter might feature grazing over several infectious sites or, otherwise, on one former carcass site carrying the different genotypes from the body fluids of the dead animal [20,40]. The test was sequentially attempted to detect *B. anthracis* through blood agar culture and Gram staining, which was then affirmed by multiplex PCR [17]. Tests were examined using MLVA and SNPs. Nonetheless, the examination of four single-nucleotide repeat markers decided these protect into six particular genotypes giving awareness on illness transmission [18,41]. Hereditary connections and atomic qualities of 34 *B. anthracis* isolates from soil and medical samples in different areas are surveyed using the MLVA and amplified fragment length polymorphism approaches [29]. An indirect ELISA relative to recombinant protective antigen domain 1 of *B. anthracis* was created and used to identify anti-PA antibodies in cows [31]. This study provides insights into the review and analysis of publications on anthrax prevalence worldwide. According to the pooled literature, we did not find a meta-analysis on anthrax prevalence in livestock. Hence, we conducted this study.

This meta-analysis showed that the collective prevalence estimate from 1992 to 2020 was 24%. It is observed from the pooled publications (Figure-2) that the global prevalence of anthrax varies between 2% and 44%, and atmospheric temperature, soil characteristics, rainfall, and river floods contribute to the spread of the pathogen and have a profound effect on the disease [17,42,43]. However, the effects of precipitation or vegetative green-up on bacterial physiology or miniature nature to drive anthrax outbreaks are unknown [44]. The continent-wise pooled prevalence study showed that anthrax is most prevalent in Africa (29%) and least prevalent in North America (21%). As we could not find any publication from South America and only one publication from Australia related to this study, we excluded these continents in the subgroup analysis. The high prevalence of anthrax in West Uganda (Africa) was due to dry climatic conditions along with alkaline soils rich in calcium and potassium, hence leading to subsequent anthrax outbreaks, and this study showed a prevalence of 25% (95% CI, 13-40%) [13]. In the study by Ert *et al.*, anthrax prevalence was 36% (95% CI, 26-47%). The use of strain-specific SNPs for low-level and high-throughput genotyping can be an effective tool in real-world forensic and public health publications [14]. In Poland, the prevalence of *B. anthracis* is 40% (95% CI, 16-68%), which is highly heterogeneous compared with those in other European countries [15]. Anthrax has emerged as a public health hazard in Zambia, with a prevalence of 48% (95% CI, 25-64%). Flooding, rainfall pattern, temperature, and evaporation along with epidemiological factors, such as cattle population, contributed to this high prevalence [17]. Disease incidence was 44% (95% CI, 22-69%), which is directly proportional to climate change [22]. The highest prevalence in Africa may be because of the high livestock population and favorable climatic conditions prevalent in this region. The study showed a prevalence of 41% (95% CI, 31-51%) [26]. Disease prevalence in Korea (Changnyeong Province) was 26% (95% CI, 13-44%]. Subsequently, the pooled study showed that the MLVA method may be significant for adaptation to environmental conditions of Asia [29]. Due to insufficient vaccination programs and the low percentage of seropositive cattle, a recurrent anthrax outbreak was noted in the Western Region of Zambia that showed a prevalence of 18% (95% CI, 12-26%) [31]. Anthrax is highly prevalent in West Africa, which is independent of the adequacy of animal immunizations in controlling anthrax, coordination, underreporting, and restricted assets, making actualizing vaccination campaigns difficult [45]. Anthrax is prevalent in Africa, the Middle East, some Asian countries, and South America. The disease has also been detected in Turkey and is mostly seen from April to November [46]. It is a neglected ailment, and its global dissemination remains under characterized. An estimated 1.8 billion individuals live within the anthrax suitable areas worldwide, an enormous number of whom live in rural areas in Africa, Europe, and Asia [47]. High-risk areas comprise 1.1 billion livestock (95% CI: 404 million-2.3 billion), with 268.1 million cattle (95% CI: 87.4-639 million), 320 million sheep (95% CI: 138-622 million), 211.2 million goats (95% CI: 74.8-453 million), 294.9 million pigs (95% CI: 103-583 million), and 0.6 million buffalo (95% CI: 0.16-1.6 million) [46]. It is predictable from the overall pooled data that the prevalence of anthrax in livestock might continue to extend in the future unless we manage it effectively by adopting necessary arrangements.

As the meta-analysis included only published articles, it may have overestimated the actual effect level. The derived results are susceptible to the methodological quality of the articles included in the study.

## Conclusion

This study estimated the pooled prevalence of anthrax worldwide in livestock using a systematic review and meta-analysis. As far as we could know, this is the first meta-analysis on the prevalence of anthrax in livestock from a global perspective. The prevalence rate of anthrax in Africa, Asia, and Europe was high, whereas that in North America was low. Hence, efficient early diagnostic strategies and scientific management practices should be implemented in high-risk regions with active involvement by veterinarians and information technologists to decrease the effects of infection and subsequently improve the economic status.

## Authors’ Contributions

BS: Collected the data, conducted the research work and wrote the manuscript. SS: Performed review and supervision. KPS: Guided the research steps. GL: Performed writing review and editing. SSP: Performed writing review and editing. GS: Performed visualization. All authors read and approved the final manuscript.
